# Validation of reference genes for quantitative RT-PCR studies of gene expression in perennial ryegrass (*Lolium perenne *L.)

**DOI:** 10.1186/1471-2199-11-8

**Published:** 2010-01-20

**Authors:** Julia M Lee, John R Roche, Danny J Donaghy, Anthony Thrush, Puthigae Sathish

**Affiliations:** 1 DairyNZ Ltd., Private Bag 3221, Hamilton 3240, New Zealand; 2 University of Tasmania, PO Box 3523, Burnie, Tasmania 7320, Australia; 3 Roche Diagnostics NZ Ltd., PO Box 62089, Mt Wellington, Auckland 1641, New Zealand; 4 Pastoral Genomics, c/o ViaLactia Biosciences (NZ) Ltd., PO Box 109185, Newmarket, Auckland 1149, New Zealand

## Abstract

**Background:**

Perennial ryegrass (*Lolium *perenne L.) is an important pasture and turf crop. Biotechniques such as gene expression studies are being employed to improve traits in this temperate grass. Quantitative reverse transcription-polymerase chain reaction (qRT-PCR) is among the best methods available for determining changes in gene expression. Before analysis of target gene expression, it is essential to select an appropriate normalisation strategy to control for non-specific variation between samples. Reference genes that have stable expression at different biological and physiological states can be effectively used for normalisation; however, their expression stability must be validated before use.

**Results:**

Existing Serial Analysis of Gene Expression data were queried to identify six moderately expressed genes that had relatively stable gene expression throughout the year. These six candidate reference genes (eukaryotic elongation factor 1 alpha, eEF1A; TAT-binding protein homolog 1, TBP-1; eukaryotic translation initiation factor 4 alpha, eIF4A; YT521-B-like protein family protein, YT521-B; histone 3, H3; ubiquitin-conjugating enzyme, E2) were validated for qRT-PCR normalisation in 442 diverse perennial ryegrass (*Lolium perenne *L.) samples sourced from field- and laboratory-grown plants under a wide range of experimental conditions. Eukaryotic EF1A is encoded by members of a multigene family exhibiting differential expression and necessitated the expression analysis of different eEF1A encoding genes; a highly expressed eEF1A (h), a moderately, but stably expressed eEF1A (s), and combined expression of multigene eEF1A (m). NormFinder identified eEF1A (s) and YT521-B as the best combination of two genes for normalisation of gene expression data in perennial ryegrass following different defoliation management in the field.

**Conclusions:**

This study is unique in the magnitude of samples tested with the inclusion of numerous field-grown samples, helping pave the way to conduct gene expression studies in perennial biomass crops under field-conditions. From our study several stably expressed reference genes have been validated. This provides useful candidates for reference gene selection in perennial ryegrass under conditions other than those tested here.

## Background

Perennial ryegrass (*Lolium perenne *L.) is the predominant grass for temperate pastoral production globally, with its popularity largely attributed to its ability to grow large amounts of high quality feed for livestock. Nevertheless, there are some limitations to perennial ryegrass, including distinct seasonal growth and quality trends [1,2]. While the environmental conditions implicated as variables affecting growth are out of farmers' control, other factors have a substantial influence on perennial ryegrass growth, for example defoliation management.

The effect of different defoliation regimes on growth have been evaluated [3-6], but the complex biological processes affected within the plant are largely unknown. Monitoring alterations in gene expression patterns facilitates our understanding of these biological processes. Traditionally, the factors that influence plant gene expression have been determined under controlled conditions, while varying one or more factors at a time [7]. Although critical to understanding gene function, these results must be validated in the field, where interactions between weather, farm management, pest challenge, and other confounding factors are likely to affect the pattern and/or degree of response [8,9].

Quantitative reverse transcription-polymerase chain reaction (qRT-PCR) is among the best methods available for determining changes in gene expression, because of its ability to quantify target genes rapidly and accurately, even those with very weak expression levels (detection limits as sensitive as one transcript per 1000 cells; [10]). Before analysis of target gene expression, it is essential to select an appropriate normalisation strategy to control for non-specific variation between samples. Introduction of inter-sample variation can occur at a number of stages throughout the experimental protocol, and can affect efficiencies of the reverse transcription (RT) and polymerase chain reaction (PCR) reactions [11,12]. The most commonly applied approach for normalisation for qRT-PCR is the use of one or more endogenous reference genes [13]. While an ideal reference gene would be universally valid, with a constant expression profile across all possible tissues and experimental conditions [12,14], no such universal reference gene has yet been reported [14-16], and is unlikely to exist [17]. However, most experimental designs are restricted to a few different tissue types or treatments, and it is likely, therefore, that one or more genes will be stably expressed across a limited experimental design.

In the past, genes that had putative housekeeping roles in basic cellular processes were frequently used as reference genes [18-20], but often without proper validation of their expression stability. Such an oversight can be misleading, as their expression has been reported to fluctuate in some instances [16,17,19]. Studies that fail to use appropriate reference genes may bias gene expression profiles and result in low precision or misleading results [11,12,14].

The aim of the current study was to identify moderately expressed genes that had relatively stable gene expression throughout the year using existing Serial Analysis of Gene Expression (SAGE™) data [21]. The stability in expression of these candidate reference genes was then validated in 442 diverse perennial ryegrass samples, grown under both field and laboratory conditions, and comprising replicated samples from different tissues/cultivars/growth stages and treatments. Expression stability was evaluated using the statistical algorithms, geNorm [17] and NormFinder [22].

## Results

### Identification of reference gene candidates

From existing SAGE™ data [[21]; constructed using the field-grown seasonal samples described in the methods section], SAGE™ tags that were mapped correctly, annotated, and had moderate expression profiles across seasons (mean of 5-50 copies per virtual ryegrass shoot cell; i.e. similar expression levels to future target genes) were identified. From the list of the SAGE™ tags that met the above criteria, six genes involved at the pre-transcription stage (histone 3, H3), transcription (YT521-B-like protein family protein, YT521-B), translation (eukaryotic translation initiation factor 4 alpha, eIF4A), and in protein biosynthesis (eukaryotic elongation factor 1 alpha, eEF1A), modification (ubiquitin-conjugating enzyme, E2) and degradation (TAT-binding protein homolog 1, TBP-1) were selected as candidate reference genes.

As eEF1A is often identified as a stable reference gene [11,23-27], a more highly expressed eEF1A gene, eEF1A (h), was also selected from the SAGE™ data for testing, along with a strategy proposed by Martin et al. [25] in which transcripts from multigene eEF1A, eEF1A (m), would be identified. The seasonal expression profile and accession numbers of the candidate reference genes from the SAGE™ data are presented in Table 1.

**Table 1:** The Serial Analysis of Gene Expression (SAGE™) tags and normalised copy numbers for candidate reference genes found in field-grown perennial ryegrass tissue sourced from pre- and post-grazed swards.

Gene abbreviation	Gene name	Accession numbers		SAGE™ tag^3^	Copies per 100,000 transcripts			
				
		dbEST^1^	TSA^2^		Winter	Spring	Summer	Autumn
eEF1A (h)	Eukaryotic elongation factor 1 alpha	GO924753	N/A^4^	CTATGTTCGA	68	87	161	102

eEF1A (s)	Eukaryotic elongation factor 1 alpha	GO924806, GO924801, GO924766, GO924798, GO924804	EZ421973	CTATGTTCGG	41	47	37	31

TBP-1	26S proteasome regulatory subunit 6A homolog	GO924783, GO924768, GO924761, GO924782, GO924758, GO924762	EZ421974	ATAATATGAA	11	13	43	29

eIF4A	Eukaryotic initiation factor 4 alpha	GO924770	N/A	TAAAACACTG	14	17	0	8

YT521-B	YT521-B-like family protein	GO924780, GO924796, GO924779, GO924805, GO924799, GO924765, GO924767	EZ421977	GAAGGTGGCT	20	10	6	2

H3	Histone 3	GO924769, GO924763, GO924764	EZ421975	AACTACTAAT	16	7	6	6

E2	Ubiquitin-conjugating enzyme	GO924794, GO924791	EZ421976	ATTTGGTTGA	5	13	0	2

^1^National Centre for Biotechnology Information GenBank dbEST accession number/s for the perennial ryegrass sequence/s to which the tags are mapped.

^2^National Centre for Biotechnology Information GenBank TSA accession number for the perennial ryegrass sequence to which the tags are mapped.

^3^Tags are presented as a 10 base pair sequence, excluding the *Nla*III site [CATG; [21]].

^4^Not applicable.

### Expression levels of the reference gene candidates

**Figure 1:**
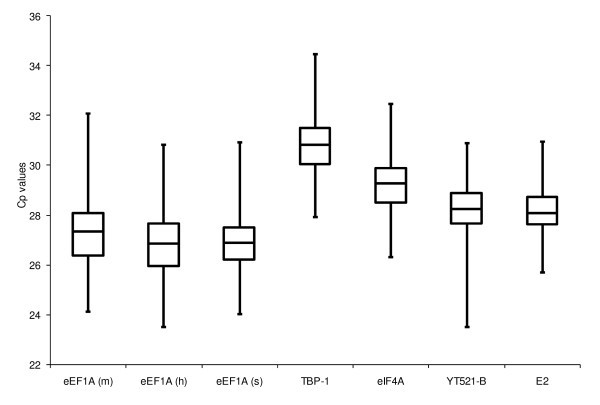
**Crossing point (Cp) value variability in candidate reference gene comparisons for the 442 perennial ryegrass samples**. Variability is displayed as medians (lines), 25^th ^percentile to the 75^th ^percentile (boxes) and ranges (whiskers).

All of the candidate reference genes were moderately abundant (median crossing point [Cp] values 26-31; Figure 1), with the exception of H3. Expression of H3 was generally very low in the tested samples (Cp values averaging 33.09 ± 2.51) or was not detected at all (n = 110 samples); therefore it was excluded from further analyses. The least variation in gene expression across all 442 tested samples was displayed by E2 (< 6 cycles), while eEF1A (m) was the most variable (8 cycles). Mean ± standard deviation of Cp values of the candidate reference genes from different tissue/cultivar/growth stage/treatment combinations are presented in Additional file 1.

### Expression stability of the reference gene candidates

**Figure 2:**
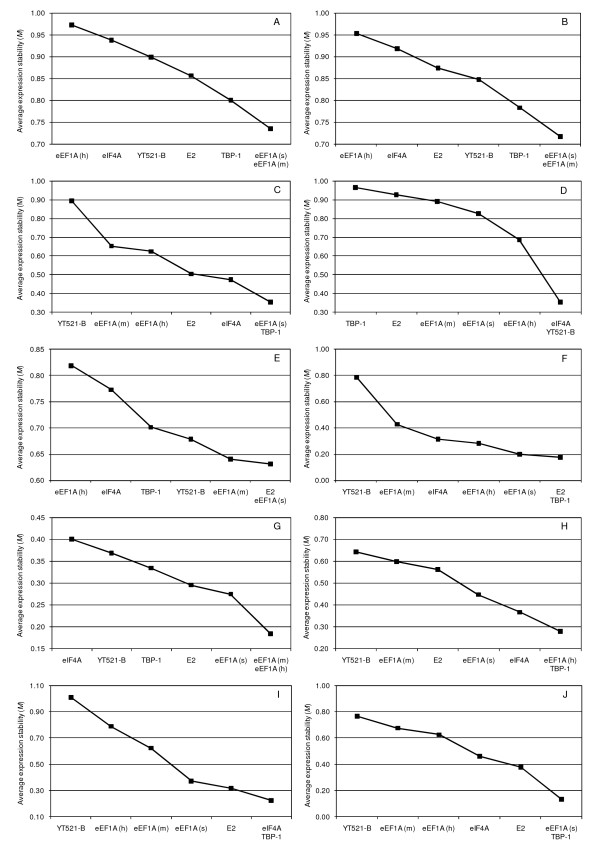
**Expression stability and ranking of candidate reference genes as calculated by geNorm**. (A) all 442 perennial ryegrass tissue samples, (B) 422 field-grown samples harvested following different defoliation management, (C) 13 laboratory-grown samples, (D) 218 perennial ryegrass stubble samples, (E) 220 perennial ryegrass leaf samples, (F) four perennial ryegrass callus, inflorescence and root samples, (G) five perennial ryegrass etiolated seedlings of different cultivars, (H) four field-grown samples harvested at the peak of each season, (I) three laboratory-grown samples to evaluate water stress and (J) four laboratory-grown samples to evaluate cold stress. A lower value of average expression stability (*M*) indicates more stable gene expression.

Data were segregated into 10 different datasets for evaluation of gene expression stability using geNorm and NormFinder (Table 2). In geNorm, when all 442 perennial ryegrass samples were included (see Table 2 for sample information), the average expression stability (*M*) of the moderately expressed eEF1A (s) and multigene eEF1A (m) was least, and that of the more highly expressed eEF1A (h), was greatest. This suggests that expression of eEF1A (s) and eEF1A (m) was most stable and eEF1A (h) was least stable (Figure 2A). This was also the case in the dataset containing the 422 field-grown leaf and stubble samples collected at different growth stages following a range of defoliation treatments (Figure 2B). Average expression stability and ranking of the candidate reference genes in the remaining datasets are presented in Figures 2C-J.

**Table 2:** The 442 perennial ryegrass samples analysed during the study and which datasets they were included in.

Experiment	Tissue type	Number of treatments	Biological replicates	Sampling dates	Total number of samples (treatments × replicates × dates)	Datasets included in^1^
Defoliation management	Leaf	6	9 (3 spatial × 3 temporal)	4	206^2^	A, B, E
	Stubble	6	9 (3 spatial × 3 temporal)	4	216	A, B, D
Cultivar	Leaf	5	1	1	5	A, C, E, G
Seasonal^3^	Leaf	4	1	1	4	A, E, H
Moisture-stress	Leaf	3	1	1	3	A, C, E, I
Cold-stress	Leaf	2	1	1	2	A, C, E, J
	Stubble	2	1	1	2	A, C, D, J
Other	Inflorescence	1	1	2	2	A, F
	Roots	1	1	1	1	A, F
	Callus	1	1	1	1	A, C, F

**Total number of samples^4^**					**442**	

^1^Datasets consist of (A) all 442 perennial ryegrass tissue samples, (B) 422 field-grown samples harvested following different defoliation management, (C) 13 laboratory-grown samples, (D) 218 perennial ryegrass stubble samples, (E) 220 perennial ryegrass leaf samples, (F) four perennial ryegrass callus, inflorescence and root samples, (G) five perennial ryegrass etiolated seedlings of different cultivars, (H) four field-grown samples harvested at the peak of each season, (I) three laboratory-grown samples to evaluate water stress and (J) four laboratory-grown samples to evaluate cold stress.

^2^There were 216 leaf samples in total, but in 10 of the samples taken immediately after defoliation there was insufficient leaf for RNA extraction.

^3^The four seasonal samples (autumn, winter, spring and summer) each consisted of two original tissue samples (one collected pre-grazing and one collected post-grazing at the peak of each season) that were bulked together after cDNA synthesis.

^4^Each of the 442 samples was tested in triplicate using qRT-PCR.

**Figure 3:**
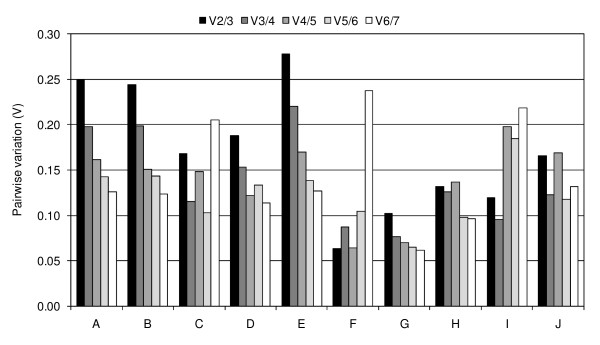
**Pairwise variation (V) to determine the optimal number of reference genes for accurate normalisation**. (A) all 442 perennial ryegrass tissue samples, (B) 422 field-grown samples harvested following different defoliation management, (C) 13 laboratory-grown samples, (D) 218 perennial ryegrass stubble samples, (E) 220 perennial ryegrass leaf samples, (F) four perennial ryegrass callus, inflorescence and root samples, (G) five perennial ryegrass etiolated seedlings of different cultivars, (H) four field-grown samples harvested at the peak of each season, (I) three laboratory-grown samples to evaluate water stress and (J) four laboratory-grown samples to evaluate cold stress.

The geNorm algorithm also calculated the pairwise variation V_n_/V_n+1_, which measured the effect of adding further reference genes on the normalisation factor, thus determining the optimal number of reference genes. Evaluation of all plant samples revealed a large decrease in the pairwise variation with the inclusion of a third, and then fourth reference gene (i.e. the differences between V2/3 and V3/4, and V3/4 and V4/5 in dataset A). When a fifth reference gene was added, the V values dropped below the proposed guideline of 0.15 (Figure 3). Thus, according to geNorm, five reference genes (eEF1A (s), eEF1A (m), TBP-1, E2 and YT521-B) are required for accurate normalisation. Pairwise variation within the field-grown samples collected at different growth stages following a range of defoliation treatments indicate that the four most stably expressed reference genes (eEF1A (s), eEF1A (m), TBP-1, and YT521-B) should be used for normalisation, while in the laboratory-grown samples use of the three most stable genes (eEF1A (s), TBP-1, and eIF4A) is sufficient (Figure 3).

The results of the NormFinder analysis are summarised in Table 3. When all 442 samples were included (dataset A), eEF1A (s) was identified as the most stable gene with an expression stability of 0.331, followed by YT521-B (0.429); eEF1A (h) was least stable (0.581). Out of the ten datasets, eEF1A (s) was most often identified as the most stable reference gene, while YT521-B was most often identified as the least stable reference gene.

**Table 3:** Stability values of candidate reference genes as calculated by NormFinder in datasets A-J^1^.

Gene	A	B	B^2^	C	D	E	F	G	H	I	J
eEF1A (m)	0.435	0.435	0.052	0.348	0.420	0.323	0.415	0.126	0.402	0.590	0.508
eEF1A (h)	0.581	0.572	0.068	0.433	0.504	0.532	0.352	0.130	0.316	0.890	0.531
eEF1A (s)	0.331	0.326	0.039	0.249	0.357	0.325	0.191	0.089	0.395	0.141	0.363
TBP-1	0.517	0.505	0.060	0.333	0.588	0.441	0.060	0.262	0.093	0.138	0.279
eIF4A	0.504	0.511	0.061	0.159	0.545	0.476	0.109	0.288	0.212	0.080	0.169
YT521-B	0.429	0.376	0.045	0.985	0.443	0.374	1.149	0.278	0.445	1.042	0.622
E2	0.539	0.540	0.064	0.396	0.501	0.330	0.060	0.176	0.343	0.288	0.153
											
Best gene/s	eEF1A (s)	eEF1A (s)	eEF1A (s)	eIF4A	eEF1A (s)	eEF1A (m)	TBP-1	eEF1A (s)	TBP-1	eIF4A	E2
Worst gene	eEF1A (h)	eEF1A (h)	eEF1A (h)	YT521-B	TBP-1	eEF1A (h)	YT521-B	eIF4A	YT521-B	YT521-B	YT521-B
											
Best two genes			eEF1A (s)/YT521-B								
Stability value			0.030								

^1^Datasets consist of (A) all 442 perennial ryegrass tissue samples, (B) 422 field-grown samples harvested following different defoliation management, (C) 13 laboratory-grown samples, (D) 218 perennial ryegrass stubble samples, (E) 220 perennial ryegrass leaf samples, (F) four perennial ryegrass callus, inflorescence and root samples, (G) five perennial ryegrass etiolated seedlings of different cultivars, (H) four field-grown samples harvested at the peak of each season, (I) three laboratory-grown samples to evaluate water stress and (J) four laboratory-grown samples to evaluate cold stress.

^2^NormFinder analysis carried out using the group property to identify the six different defoliation treatments contained within this dataset. As well as identifying the best reference gene; this analysis gives the combination of the two best reference genes with their combined stability value.

NormFinder has the added ability of being able to estimate the variation between sample groups or treatments (as described further in the methods section). This function determines the best combination of two reference genes for normalisation. It also establishes whether normalisation using the two reference genes in combination will be more accurate than just using the most stable gene (i.e. if the stability value of the best two gene combination is lower than that of the most stable gene).

The dataset containing the 422 field-grown leaf and stubble samples collected at different growth stages following a range of defoliation treatments was the only dataset that contained sufficient replication to allow this full analysis (see Table 2 for replicate information). Analysing this dataset with or without the treatment groups identified did not affect the ranking of the genes, although the stability values were reduced with the inclusion of treatment groups (Table 3). With the treatment groups identified in this dataset, NormFinder selected eEF1A (s) as the most stably expressed single gene, with a stability value of 0.039. The best combination of two genes, eEF1A (s) and YT521-B, further reduced the NormFinder stability value to 0.030.

### Comparison of reference genes for normalisation of a target gene

The expression levels of a target gene, chloroplast translational elongation factor Tu (EF-Tu, GenBank dbEST accession number GR517729), were used as an example to show the effect of using different reference genes for normalisation. The EF-Tu expression was normalised using three different strategies: 1) geometric average of the four most stably expressed reference genes selected by geNorm, 2) geometric average of the two most stably expressed reference genes selected by NormFinder, and 3) the least stably expressed gene according to both geNorm and NormFinder used alone.

**Figure 4:**
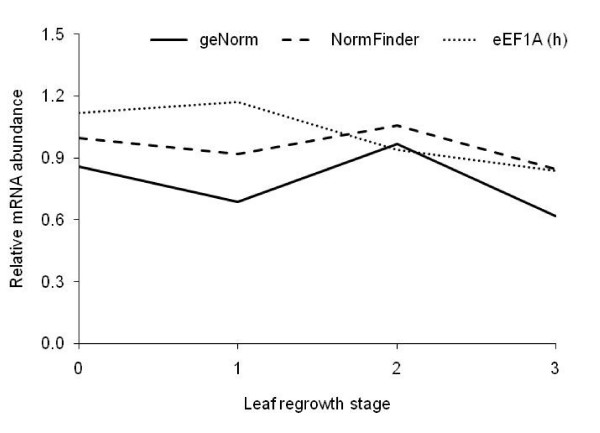
**Relative quantification of the target gene EF-Tu in perennial ryegrass leaf tissue throughout regrowth**. Normalisation was carried out using the four most stable reference genes defined by geNorm, the two most stable reference genes defined by NormFinder or the least stable reference gene, eEF1A (h).

In perennial ryegrass leaf tissue, there was no effect (*P *> 0.1) of defoliation frequency or severity, or any interaction between the defoliation treatments. There was, however, a significant (*P *< 0.001) interaction between leaf regrowth stage and the normalisation strategy used. Normalisation using the least stable reference gene (eEF1A (h)) led to over-estimation of the target gene following defoliation (0-leaf stage) and at the 1-leaf stage of regrowth compared with the geNorm strategy, and at the 1-leaf stage of regrowth compared with NormFinder (Figure 4). Although the geNorm and NormFinder strategies did differ in their estimation of the target gene at the 1- and 3-leaf stages of regrowth, the trend in transcript abundance throughout regrowth remained the same, in contrast with that displayed following normalisation using eEF1A (h).

## Discussion

Quantitative RT-PCR has become a powerful tool for analysis of gene expression because of its high throughput, sensitivity, and accuracy [14]. However, the use of one or more stably expressed reference genes to normalise the variation introduced by RNA sample quality, RNA input quantity, and RT enzymatic efficiency is essential to achieving reliable results [13,17]. To obtain a solid basis for normalisation of gene expression data, it is advisable to validate the expression stability of candidate reference genes under the conditions studied, rather than using reference genes published elsewhere [28]. Validation of reference genes has been simplified with the design of statistical algorithms, such as geNorm and NormFinder, which not only test the expression stability of reference genes, but can also determine the number of reference genes required to provide accurate normalisation [17,22].

This study describes the validation of candidate reference genes for normalisation of gene expression in perennial ryegrass. The most comprehensive dataset contains 422 field-grown leaf and stubble samples collected at different growth stages following a range of defoliation treatments and representing spatial and temporal replicates. Using geNorm, eEF1A (s) and eEF1A (m) were identified as the two most stable genes across the wide range of samples tested, followed by TBP-1 and YT521-B. Use of all four of these genes is recommended for normalisation, based on a suggested pairwise variation threshold of 0.15 [17]. However, this is not an absolute rule and depends on the data. In the current study, based on V ≤ 0.15, two reference genes are sufficient for normalisation of qRT-PCR data from the callus, inflorescence and root samples, the etiolated seedlings of different cultivars, the laboratory-grown samples to evaluate water stress, and the field-grown samples harvested at the peak of each season.

One of the factors that may have made it more difficult to achieve V ≤ 0.15 in the current study is the large number of samples and treatments tested. Datasets containing smaller numbers of samples and treatments tended to require fewer reference genes for accurate normalisation [28] than larger datasets (> 50 samples; 2-4 treatments). As far as we are aware, the maximum number of qRT-PCR samples analysed hitherto using geNorm was 91 samples [29]. Moreover, even other studies that analysed smaller sample numbers (< 50) were not able to obtain V values smaller than 0.21 when they tested eight or ten candidate reference genes in human breast cancer and osteoarthritic cartilage samples [30,31]. Perennial ryegrass, like the human species, is an outcrossing, heterogenous species; hence within a sample of the ryegrass population there can be considerable genetic diversity between plants [32,33]. Thus, more reference genes may be required to stabilise the variability in gene expression. The larger threshold for variability in reference gene expression may have also been a result of the fact that the plants were field-grown, and thus exposed to constantly changing environmental conditions.

In slight contrast to geNorm, the alternative algorithm, NormFinder, ranked eEF1A (s), YT521-B, eEF1A (m), and TBP-1 as the four most stably expressed genes in this dataset, with eEF1A (s) and YT521-B providing the best combination of two genes for normalisation of gene expression data. Although the reduction in the stability value when using the single, most stably expressed gene (0.039) compared with the two most stably expressed (0.030) is not large, if small differences in gene expression are to be detected then this increase in the accuracy of normalisation is still desirable.

Some studies that have utilised both geNorm and NormFinder have reported minor changes in gene stability ranking [26,34-36], while others have observed relatively substantial changes [i.e. up to 15 places between the two methods; [37-39]. GeNorm and NormFinder rely on different mathematical approaches to calculate stability. GeNorm selects two genes with a low intra-group variation and approximately the same non-vanishing inter-group variation. In comparison, NormFinder selects the two best genes with minimal combined inter- and intra-group expression variation [22], which can have a notable effect on the subsequent gene stability ranking [40]. Therefore, the fact that the ranking of candidate reference genes by NormFinder is not always identical to that defined by geNorm is not surprising. Some studies have chosen to base their reference gene selection on geNorm results because of its ability to identify the appropriate number of reference genes for accurate normalisation [37,41]. Others have chosen NormFinder, because it examines the stability of each single reference gene independently, and not in relation to the other genes as geNorm does; which is important considering our limited knowledge regarding gene co-regulation [38].

When the target gene EF-Tu was quantified using the four reference genes recommended by geNorm, the two suggested by NormFinder or the least stable gene there were some differences in the calculated transcript abundance. The geNorm and NormFinder strategies differed in their estimation of EF-Tu at the 1- and 3-leaf stages of regrowth. In an ideal situation, normalisation using the genes defined by geNorm or NormFinder would have produced exactly the same result, indicating that there was no additional benefit in using four reference genes as opposed to two. While this wasn't the case, it is difficult to say whether the mathematical approach used by geNorm or NormFinder is superior. Therefore, the fact that the trend in transcript abundance throughout regrowth remained the same for both strategies suggests that either approach could be used for normalisation. Both approaches demonstrate the same up- or downregulation of the target gene, it is just the magnitude of the effect that differs, which could be taken into consideration when interpreting results. The trend in transcript abundance produced from normalisation using the least stable gene differed to the other two strategies. This highlights the importance of validating reference gene stability to ensure that low precision or misleading results do not occur [11,12,14].

Some of the reference genes used in the current study have been used previously as reference genes in other species. In plant studies, eEF1A is probably the most commonly used reference gene [42-44], although its expression profile has not always been validated before use. Using geNorm and/or NormFinder, eEF1A has been ranked as one of the top two reference genes in rice, perennial ryegrass, potato, and *Lolium temulentum *[23-25,45], and third best in Arabidopsis *(Arabidopsis thaliana *L.), *Brachypodium distachyon*, and *Ectocarpus siliculosus *[11,26,27]. However, while eEF1A was proven to be a good reference gene in the current, and other published studies, there is also evidence that its expression profile is not as consistent as that of other reference genes that have been tested [18,19,39,40]. One possible reason for this is that eEF1A is encoded by several genes in a multigene family, members of which have been shown to exhibit differential expression [46,47]. In the current study the gene expression of two individual eEF1A genes in perennial ryegrass was analysed. The moderately expressed eEF1A (s) had a more stable expression profile than the more highly expressed eEF1A (h). This difference in the expression stability may explain why contrasting results exist for many tested reference genes [18,24,46], and highlights the importance of validating expression stability of candidate reference genes before use for normalisation.

Brunner et al. [12] suggested that simultaneous amplification of two or more members of a reference gene family by a single primer pair designed in the conserved coding region could result in more stable gene expression than a single gene amplification. This assumes that genes within the same family will have balanced expression, which is not always the case, as demonstrated with eEF1A here and by Reid et al. [48], with ubiquitin by Jain et al. [24], and with actin by Jian et al. [18].

The reference gene YT521-B, although identified as the most unstable gene in half of the datasets, was selected by NormFinder, along with eEF1A (s), as providing the best two-gene combination for normalisation of gene expression data in the most comprehensive dataset. To our knowledge YT521-B has not been tested before for use as a reference gene. In Arabidopsis, Bläsing et al. [49] illuminated five-week old seedlings for 4 hours in the presence of ambient or < 50 ppm carbon dioxide (close to compensation point where the rates of photosynthesis and respiration are balanced). They identified an YT521-B-like family protein (At5 g61020; orthologous to YT521-B from the current study) as a carbon fixation-responsive gene, which perhaps makes it slightly surprising that it was considered to be a stably expressed gene in the field-grown samples collected following a range of defoliation treatments. However, in eukaryotic cells, YT521-B are said to be ubiquitously expressed nucleolar proteins [50] playing vital roles in the assembly of genes into transcription centres and allowing efficient gene expression regulation [51]. YT521-B appears to be a good reference gene candidate; however it is vital that it is validated due to its instability under some experimental conditions.

To the best of our knowledge, no other study has attempted to analyse gene expression in field-grown perennial monocotyledons, let alone perennial ryegrass. To successfully commercialise perennial plants specific for pastoral/turf/biofuel use, an understanding of gene expression in these plants during their regrowth cycle is necessary before we can harness the power of biotechnology for various industries. The prelude to this would be to validate a set of reference genes in order to harmonise the data from various experiments that are expected to follow suit. We believe that we have achieved this by the validation of several suitable reference genes for normalisation of target genes involving not only perennial ryegrass plants raised in controlled conditions, but also in the field.

## Conclusions

This study is unique in the magnitude of samples tested with the inclusion of numerous field-grown samples, helping pave the way to conduct gene expression studies in perennial biomass crops under field-conditions. Our results indicate that eEF1A (s) and YT521-B are suitable reference genes for normalisation of target genes in perennial ryegrass following different defoliation management in the field. Several other stably expressed genes have also been validated providing useful guidelines for reference gene selection in perennial ryegrass under conditions other than those tested here.

## Methods

### Plant material and growth conditions

#### Seasonal field-grown samples

Field-grown samples of diploid perennial ryegrass (cv. Bronsyn) were collected from livestock-active perennial ryegrass dominant paddocks at DairyNZ's Lye Farm in Hamilton, New Zealand (37°47'S 175°19'E; elevation 40 m above sea level). Tissue samples, comprising mainly viable leaves, were collected at midday during the peak of each season (autumn, winter, spring and summer) from autumn 2003 to summer 2004 pre- and post-grazing, resulting in four pre-grazing samples and four post-grazing samples. For full experimental details refer to Sathish et al. [21]. Samples were frozen immediately in liquid nitrogen, transported in dry ice, and stored at -80°C before RNA extraction.

#### Field-grown samples under different defoliation management

At the same farm in April 2007, 54 plots (each 2 × 3 m) were laid out in a newly-mown diploid perennial ryegrass (cv. Bronsyn) dominant sward. Defoliation treatments were allocated to the plots in a randomised block design. Treatments consisted of two defoliation frequencies (when either one or three new leaves per perennial ryegrass tiller had fully expanded, i.e. 1- or 3-leaf regrowth stage) and three defoliation severities (defoliation to either 20, 40, or 60 mm residual stubble height) compared in a 2 × 3 factorial arrangement. Each treatment was replicated nine times, comprising three spatial replicates sampled over three temporal replicates. The spatial replicates of each treatment were defoliated on the same date, with the second and third groups defoliated three and seven days after the first group, respectively.

In late June 2007, the groups were harvested to 40 mm residual stubble height using a rotary lawnmower as above. Defoliation frequency treatments commenced from this point, with 27 plots defoliated three times at the 1-leaf regrowth stage (frequently; 19 July, 3 August, and 20 August for group one, with the second, and third groups harvested three and seven days later, respectively). The remaining 27 plots were defoliated once at the 3-leaf regrowth stage (infrequently), which coincided with the third 1-leaf stage harvest (20 August for group one, with the second and third groups harvested three and seven days later, respectively). At this harvest, all plots were defoliated to their respective treatment residual stubble height (20, 40, or 60 mm).

On the day following the final treatment defoliation in August 2007, and again following the emergence of each successive full new leaf on perennial ryegrass tillers (i.e., at the 1-, 2-, and 3-leaf stages of regrowth), viable samples (approximately 5 g fresh weight) of both perennial ryegrass leaf and stubble tissue were collected at random from each plot. Stubble was defined as the heterogeneous plant compartment that includes both fully expanded leaf material (leaf sheaths), as well as basal immature parts of expanding leaves or elongating leaf bases [52,53]. Samples were collected at midday, using a scalpel to cut individual tillers from different plants at ground level. Care was taken not to include dirt, floral stems, or dead/diseased material in the sample. The tissues were frozen immediately in liquid nitrogen, transported in dry ice, and stored at -80°C before RNA extraction.

#### Field-grown root and inflorescence samples

At the same farm in October 2008, tillers from multiple different diploid perennial ryegrass (cv. Bronsyn) plants were collected at midday from a perennial ryegrass dominant sward, this time including root tissue. Inflorescent tissue that had not yet emerged from reproductive tillers was collected and bulked based on the length (i.e. maturity) of the inflorescence (<40 or >40 mm length, INF<40 and INF>40 respectively). Samples were frozen immediately in liquid nitrogen, transported in dry ice, and stored at -80°C before RNA extraction. Root tissue was also removed from the base of both vegetative and reproductive tillers, washed to remove dirt, frozen immediately in liquid nitrogen after washing and stored at -80°C before RNA extraction.

#### Laboratory-grown callus tissue

For full experimental details on calli induction see Bajaj et al. [54]. Briefly, the meristematic region of laboratory-grown perennial ryegrass tillers (cv. Impact) were cut and spread on Murashige and Skoog (MS) medium [55] supplemented with 3% (w/v) sucrose, 22.6 μM 2,4-dichlorophenoxyacetic acid (2,4-D), and cultured in the dark for four weeks at 24 ± 2°C. Calli induced from these tissues were sub-cultured once for two weeks in the dark on MS medium supplemented with 3% (w/v) sucrose, 9 μM 2,4-D and 0.44 μM benzyl adenine, and then sub-cultured once again for 5-7 days. Calli were harvested, frozen immediately in liquid nitrogen, and stored at -80°C before RNA extraction.

#### Laboratory-grown etiolated seedlings

Perennial ryegrass seeds from diploid (cv. Aries, Banquet, Bronsyn, and Impact) and tetraploid cultivars (cv. Quartet) were sown on moist filter paper placed in petri-plates and germinated in darkness for 10 days at 22°C day/18°C night temperature and 80% relative humidity (RH). On the 11^th ^day the seed was trimmed off the seedlings, and the seedlings were frozen immediately in liquid nitrogen before being stored at -80°C before RNA extraction.

#### Laboratory-grown samples to evaluate cold stress

Diploid perennial ryegrass (cv. Bronsyn) plants were grown in controlled-environment chambers under cool-white fluorescent lights. Seeds were planted at a depth of 10 mm in two pots, each measuring 125 mm diameter × 100 mm high, and filled with Yates Black Magic seed raising mix (Orica Ltd, Auckland, New Zealand) containing slow-release nutrients. Plants in both pots were kept hydrated for 104 days at 22°C day/16°C night temperatures, 16-h-light/8-h-dark cycle, and 85% RH. Within this period, plants were defoliated to approximately 80 mm residual stubble height six times (52, 62, 72, 82, 92, and 102 days after the seeds were sown).

Experimental conditions were then applied as follows: one pot of control plants were grown for 10 days at 22°C day/16°C night temperatures, 16-h-light/8-h-dark cycle, and 85% RH under irrigation; the second pot of plants were grown for 10 days at 6°C day/4°C night temperatures, 16-h-light/8-h-dark cycle, and 70% RH. On the 11^th ^day samples of leaf and stubble were collected from both the control and cold treatments, frozen immediately in liquid nitrogen and stored at -80°C before RNA extraction.

#### Laboratory-grown samples to evaluate moisture-stress

Diploid perennial ryegrass (cv. Bronsyn) plants were grown in controlled-environment chambers under cool-white fluorescent lights. Seeds were planted at a depth of 10 mm in two pots, each measuring 125 mm diameter × 100 mm high, and filled with Yates Black Magic seed raising mix (Orica Ltd, Auckland, New Zealand) containing slow-release nutrients. Plants in both pots were kept hydrated for 73 days at 20°C day/18°C night temperatures, 16-h-light/8-h-dark cycle, and 85% RH. Within this period, plants were defoliated to approximately 80 mm residual stubble height three times (52, 62, and 72 days after the seeds were sown).

Experimental conditions were then applied as follows: one pot of control hydrated plants was grown for 7 days at 22°C day/16°C night temperatures, 16-h-light/8-h-dark cycle, and 70% RH under irrigation. The second pot of plants was gradually dehydrated over 3 days at 28°C day/20°C night temperatures, 16-h-light/8-h-dark cycle, and 70% RH, followed by 3 days at 28°C day/20°C night temperatures, 16-h-light/8-h-dark cycle, and 50% RH with no irrigation. Following this, the dehydrated plants were rehydrated by saturating the seedling mix with water and the plants were held for 24 h at 22°C day/16°C night temperatures, 16-h-light/8-h-dark cycle, and 70% RH. Samples of leaf tissue were collected from dehydrated plants at the end of day 6 (half the plants in the pot), and from hydrated (control) and rehydrated plants at the end of day 7. Samples were frozen immediately in liquid nitrogen and stored at -80°C before RNA extraction.

### RNA extraction and mRNA isolation

Frozen perennial ryegrass tissues (callus, etiolated seedlings, inflorescence, leaf, stubble and root) from all conditions were ground independently in liquid nitrogen. Total RNA was extracted using the RNeasy Plant Mini Kit (Qiagen, Hilden, Germany) according to the manufacturer's protocol. Residual genomic DNA was removed by on-column DNAse I digestion, using the RNase-free DNase set (Qiagen), and mRNA was purified from total RNA using Dynabeads^® ^Oligo (dT)_25 _(Invitrogen Dynal AS, Oslo, Norway). The mRNA concentration and purity were determined using a Nanodrop ND-1000 spectrophotometer (Nanodrop Technologies Inc., Wilmington, DE, USA); each mRNA sample was assayed twice and an average value determined.

Absence of genomic DNA contamination was confirmed by performing qRT-PCR on 0.1 ng of each of the mRNA samples using primers designed for the small subunit of ribulose-1,5-bisphosphate carboxylase gene (forward and reverse primers in 5'→3' direction are GAGGAGTCCGGCAAGGCATAA and TATGCTTTTACATGTAGCCGGTTC, respectively).

### First strand cDNA synthesis

Messenger RNA (10 ng) was reverse transcribed to produce cDNA using the Transcriptor First Strand cDNA Synthesis Kit (Roche Diagnostics, Mannheim, Germany) with anchored-oligo (dT)_18 _primers in total reaction volumes of 20 μl. The two cDNA samples from each season (one pre-grazed and one post-grazed) were bulked together, resulting in four seasonal samples (autumn, winter, spring and summer). All cDNA samples were diluted 100-fold with PCR-grade water.

### PCR primer design

Primer pairs were designed to amplify a large portion of the 3' untranslated region (3'UTR) of the candidate reference genes and the target gene using Primer3 software [http://primer3.sourceforge.net/; accessed 2007/2008; [56]] and are described in Table 4. To ensure maximum specificity and efficiency during PCR amplification a stringent set of criteria was used for primer design [57]. This included predicted melting temperatures (T_m_) of 58-60°C, primer lengths of 19-24 nucleotides, guanine-cytosine contents of 36-58%, and PCR amplicon lengths of 111-168 base pairs. All primers were custom-ordered from a commercial supplier (Invitrogen, Auckland, New Zealand).

**Table 4:** Primer sequences, amplicon sizes, and polymerase chain reaction (PCR) amplification efficiency for the candidate reference genes and target gene.

Reference/target gene	Gene abbreviation	Primer sequences (5' → 3')	Primer designed in	Amplicon size (bp)	Product in 3'UTR (bp)	Amplification efficiency
Reference	eEF1A (m)	(F) GGC TGA TTG TGC TGT GCT TA	Coding region	114	0	1.883 ± 0.0737
		(R) CTC ACT CCA AGG GTG AAA GC	Coding region			
Reference	eEF1A (h)	(F) ATG TCT GTT GAG CAG CCT TC	3'UTR	108	108	1.975 ± 0.0562
		(R) GCG GAG TAT ATA AAG GGG TAG C	3'UTR			
Reference	eEF1A (s)	(F) CCG TTT TGT CGA GTT TGG T	3'UTR	113	113	1.975 ± 0.0278
		(R) AGC AAC TGT AAC CGA ACA TAG C	3'UTR			
Reference	TBP-1	(F) TGC TTA GTT CCC CTA AGA TAG TGA	Coding region/3'UTR	112	105	1.861 ± 0.0302
		(R) CTG AGA CCA AAC ACG ATT TCA	3'UTR			
Reference	eIF4A	(F) AAC TCA ACT TGA AGT GTT GGA GTG	3'UTR	168	168	1.922 ± 0.0036
		(R) AGA TCT GGT CCT GGA AAG AAT ATG	3'UTR			
Reference	YT521-B	(F) TGT AGC TTG ATC GCA TAC CC	Coding region/3'UTR	122	112	1.916 ± 0.0952
		(R) ACT CCC TGG TAG CCA CCT T	3'UTR			
Reference	H3	(F) CAC CAA TGT TCT GCC TAT CG	3'UTR	135	135	1.850 ± 0.1250
		(R) CAG ACC AAC GAA CAA ACG AC	3'UTR			
Reference	E2	(F) CGG TTC TGT GCC AAA ATG T	3'UTR	111	111	1.854 ± 0.0181
		(R) CAG CTA TCT CCA ACG GTT CA	3'UTR			
Target	EF-Tu	(F) AAT GCC CAC CAT GAG AAT TT	3'UTR	137	137	1.955 ± 0.0709
		(R) ATG CAA GCA AAA CCA CTT GA	3'UTR			

### qRT-PCR conditions

The qRT-PCR were performed in 384-well plates with a LightCycler^® ^480 real-time PCR instrument (Roche Diagnostics) using the LightCycler^® ^480 SYBR Green I Master kit. The reaction set-up was performed on the epMotion^® ^5075LH automated liquid handling system (Eppendorf, Hamburg, Germany). Reactions were performed in triplicate and contained 5 μl SYBR Green I Master, 2 μl PCR-grade water, 2 μl cDNA, and 0.5 μl of each of the 10 μM forward and reverse gene-specific primers in a final volume of 10 μl. In addition, each plate contained no-template controls and two calibrator samples required for normalisation of the target gene (one leaf and one stubble sample collected at the 3-leaf stage, i.e. immediately before defoliation).

The reactions were incubated at 95°C for 5 min to activate the FastStart *Taq *DNA polymerase, followed by 45 cycles of 95°C for 10 sec, 60°C for 10 sec, and 72°C for 8 sec. The specificity of the PCR reaction was confirmed with a heat dissociation protocol (from 60°C to 95°C) following the final PCR cycle. This ensured the resulting fluorescence originated from a single PCR product, and did not represent primer dimers formed during PCR or a non-specific product. Amplification of a single product of expected size was verified by gel electrophoresis on a 1.5% agarose gel (Sigma-Aldrich, St Louis, MO, USA) and ethidium bromide staining.

LightCycler^® ^480 software (version 1.5; Roche Diagnostics) was used to collect the fluorescence data. PCR efficiencies were calculated using the equation E = 10^-1/slope ^on a standard curve generated using a tenfold dilution series of one sample (leaf and stubble) over three dilution points that were measured in triplicate.

The mean, standard deviation (SD), and coefficient of variation (CV) of the raw triplicate qRT-PCR values within each plate were determined. Samples whose CV were greater than 1.5% were inspected; a reaction was considered an outlier if one of the triplicate reactions deviated by more than 1 SD from the mean and it was excluded from analysis. Samples were repeated if exclusion of one of the reactions still did not result in a CV <1.5%.

### Determination of candidate reference gene expression stability

Two publicly available software tools, geNorm [17] and NormFinder [22] were used to evaluate gene expression stability. Both tools require the transformation of Cp values to linear scale expression quantities. Using the LightCycler^® ^software, Cp values were converted into quantities via the standard curve with the Absolute Quantification Fit Points method, and both measures were exported into Microsoft Excel.

To ensure that data from different plates were comparable, the quantities for each gene were then normalised to the quantity of the 1/100 dilution from the standard curve dilution series that was run on each plate. For example, the dilution series on the first plate for eIF4A resulted in an average quantity of 0.0102 for the triplicate 1/100 dilution. Following absolute quantification, the average quantities on the second, third and fourth plates for the triplicate 1/100 dilution were 0.0102, 0.0098 and 0.0108. Normalisation factors for each plate were calculated by dividing 0.0102 by the average quantity for each plate, resulting in normalisation factors of 1.0020, 1.0404, and 0.9501 for the second, third and fourth plates, respectively. The quantities for each of the samples on each plate were then multiplied by the calculated normalisation factor.

The quantities were then imported into the two software tools, geNorm (version 3.5) and NormFinder, which were used as described by Vandesompele et al. [17] and Andersen et al. [22], respectively. NormFinder has the added ability of being able to estimate the variation between sample groups (i.e. treatments). In most of our datasets there was not enough samples per treatment to fully utilise this function; therefore, only a simple analysis of gene stability was carried out which identified the most stable gene in each dataset. The dataset containing the 422 field-grown leaf and stubble samples collected at different growth stages following a range of defoliation treatments did however contain sufficient replication to allow a full analysis, enabling the combination of the two best reference genes to be determined.

### Normalisation of the target gene

Normalised ratios of the target gene EF-Tu in perennial ryegrass leaf tissue collected following different defoliation management were calculated from the LightCycler^® ^Relative Quantification Software (Roche Diagnostics) using the formula:

This formula provides an efficiency-corrected relative quantification, normalised to a calibrator sample (perennial ryegrass leaf at the 3-leaf stage), where TS is the concentration of the target gene in a sample, RS is the concentration of the reference gene in a sample, TC is the concentration of the target gene in the calibrator sample and RC is the concentration of the reference gene in the calibrator sample.

The EF-Tu expression was normalised using three different strategies: 1) geometric average of the four most stably expressed reference genes selected by geNorm, 2) geometric average of the two most stably expressed reference genes selected by NormFinder, and 3) the least stably expressed gene according to both geNorm and NormFinder used alone.

### Statistical analysis

The normalised ratios of the target gene in leaf tissue were log_10_-transformed before statistical analysis. Results were analysed using mixed models with a compound symmetry covariance structure for the repeated measurements through time in GenStat 11.1 [58]. The statistical model included defoliation frequency and severity, time (leaf regrowth stage), the normalisation strategy, and all possible interactions between defoliation frequency, severity, time, and strategy. Following statistical analysis, averages were back-transformed, and results expressed as the mRNA transcript abundance relative to the leaf calibrator sample.

## Authors' contributions

This study was designed with the assistance of all authors. JML performed all the sample preparation, experimental procedures, data analysis, and wrote the draft manuscript. PS supervised the study and assisted with data analysis. AT assisted with data analysis. All authors contributed to, read, and approved the final manuscript.
